# Correspondence

**Published:** 1918-03

**Authors:** F. W. McGuire


Dec. 18, 1918.

A. L. Benedict, M. D.,
Buffalo, N. Y.

Dear Sir:—

In accordance with your request, I am sending you a short account of my trip to England with Base Hospital No. 23.

After a hesitating start from Buffalo which finally occurred on Nov. 21, 1917, and, after a rather disagreeable and stormy voyage, we arrived in England about two and one-half weeks later. The busy censor prevents one from being definite and, consequently, I cannot give you as much information as I would like. But our voyage held some interest. We were unusually fortunate in having twenty-five such good fellows and I think that it would have been difficult to have selected a more agreeable and jolly aggregation. At all times there was plenty of fun and goodfellowship. The weather and sea however were different. The weather was cold, considerable rain and enough fog to make our trip more or less perilous. The sea was very rough all the way over and occasionally a vacant chair at table told us that its former occupant was Hooverizing. We travelled without outward lights at night and it was so dark and desolate that many times it was possible to be within three feet of another person and not appreciate it. I believe that it was at such times that we had the most apprehension. I personally saw no submarines except in my sleep and I believe that this fact accounts for some of the large numbers that have been seen on other voyages.

We finally arrived at a seaport "Somewhere in England" but our departure from this port was delayed, in inclement weather, for a number of hours. The delay was due to the great number of soldiers who were entraining at this point. However, when our turn came, we boarded a train for our rest camp. The English trains are very much smaller than our own and are conspicuous in their lack of comforts. They are divided into compartments and frequently are without heat.

After arriving at our rest camp I was unfortunate in being compelled to take to my bed on account of an attack of Broncho-Pneumonia. I, however, was able to be up in about twelve days but the rest of the Unit had, in the meantime, departed for their station in France and I was left behind. It is rather an interesting experience to be sick in bed while associated with an organization and have all its members depart suddenly and leave you alone and friendless in a strange country. I think the day that the unit left me stranded was the most desolate one which I have ever spent, but this is just one of the experiences which increases a man's interest in life.

The one thing very pleasing to me was the kindness extended by the English people. American soldiers are received everywhere in England with the greatest consideration and, while the Englishman at first approach may seem very diffident, after you have once come to know him well you will find that he will sacrifice a great deal of his personal reticence to be of service to you. On one occasion, in a London fog, an Englishman went three blocks out of his way in order to direct me to my hotel. Many other instances of his kindness could be mentioned.

Shortly after getting out of bed I went to London to ascertain the advisability of my going to France. When I reached headquarters, the authorities would not allow me to proceed but appointed an examining board which advised my return to America.

While awaiting orders in London I had numerous opportunities to visit the various hospitals, both civil and military, and it might be of interest to note that the civil hospitals impress one with their great size, their age, their good management, and the fact that they have no private rooms. Consequently, there are no pay cases and the hospitals are principally maintained by public subscription. All private cases are either operated at home or go to a nursing home for their treatment.

One is further impressed by the skill of the operators, their dexterity and their knowledge of anatomy, but in many instances their technique differs from ours. The anaesthetic of choice in the London hospitals is ether, and in only one or two of the hospitals did I find gas used. While there I visited a number of the military hospitals, and I believe that in their line they are doing excellent work. I was especially interested in the Froghal Jaw Hospital at Sidcup, Kent, where nothing but face and jaw cases are handled. This hospital is under the management of Major Gillies who is doing some very interesting work in the shifting of skin, especially from the head and chest to the face. At his first operation he dissects the skin and makes a tube of skin which acts as a connection for nourishment. At a later operation he shifts the skin to the desired locality with the tube still connected. Later, when he finds that the skin is going to live, he returns the tube of skin to its original place. The improvement in the facial appearance of some of these cases is wonderful. I saw one case in which an entire nose was being made. This type of operation is very difficult, because a supporting bridge must first be formed, and this is made by shifting the superior turbinate bones and placing upon them a bony and cartilaginous graft from the ninth rib. A very large graft of the skin and underlying structures is then applied and sutured into place. The graft is made purposely large to allow of contraction later. The inner surface is then epithelized and the final result is startling.

In the bone graft work the methods described by Albee are followed, using a portion of a rib for the graft material. Major Gillies also has a very interesting method of getting transplanted skin to live but, as he has not written about it up to the present time, I feel that it would be inconsiderate on my part to describe the process in print.

At this hospital there were three hundred and fifty resident patients and about the same number of non-residents. The hospital is beautifully situated in the country and is on the temporary cottage plan. The technique here was excellent, and I expect that in the very near future we will hear a mighty good report from this hospital.

From the number of injured that I saw in the military hospitals in England, it is quite evident that we will soon face the same conditions in America. Some of our boys will soon be returning to us wounded, broken down and some cripples for life. Those that are left in the United States cannot do too much in the way of contributing money and sending various little comforts to the boys who are bearing the brunt of battle in the front line trenches. The things most desired at that time were socks, American tobacco and cigarettes. As a usual thing, sweaters are contra-indicated in the front line trenches because of the vermin.

In closing I might say that one cannot help but be impressed with the fact that the British people as a whole will continue fighting until the aims of Democracy have been realized.

Sincerely yours,

F. W. McGUIRE.

